# The Bioinformatics and Experimental Analysis of CD276 for Prognosis and Immune Infiltrates in Colon Adenocarcinoma

**DOI:** 10.1002/cnr2.70503

**Published:** 2026-02-22

**Authors:** Rui Chen, Tao Zhang, Jingru Yang, Longxia Zhang, Fei Su

**Affiliations:** ^1^ Department of Oncology The First Hospital of Lanzhou University Lanzhou China; ^2^ School of Basic Medical Sciences Lanzhou University Lanzhou China

**Keywords:** CD276, clinical outcome, colon adenocarcinoma, immune infiltration, prognosis

## Abstract

**Background:**

Colon adenocarcinoma (COAD), although the third‐most common type of gastrointestinal tumors, still lacks specific biomarkers for early diagnosis, treatment, and prognosis.

**Aims:**

This study aimed to evaluate the CD276 in tumorigenesis, prognosis and immunity for colon adenocarcinoma.

**Methods and Results:**

The CD276 expression in colon adenocarcinoma was established by using RNA‐sequencing transcriptomic data of The Cancer Genome Atlas (TCGA) databases. The biological functions of CD276 were evaluated using the Metascape database and Gene Set Enrichment Analysis (GSEA). The association between CD276 and immune cell infiltration was investigated by TIMER website. Correlation analysis was performed between CD276 expression and clinicopathological characteristics. CD276 expression was significantly elevated in colon adenocarcinoma tumor (*p* < 0.0001). High CD276 was associated with microsatellite instability (MSI) status, patients' survival, and disease progression. Cox regression analysis revealed that CD276 was a risk factor for overall survival [hazard ratio (HR): 1.848, *p* = 2.64E−03], disease‐specific survival (HR: 2.406, *p* = 5.35E−04), and progression‐free interval (HR: 1.772, *p* = 2.04E−03). Moreover, CD276 level was significantly associated with tumor immune cell infiltration, biomarkers of immune cells, and immune checkpoint expression.

**Conclusions:**

Our analyses indicated that increased CD276 may contribute to colon adenocarcinoma development by activating tumor‐promoting signal pathways and altering the immune microenvironment.

**Abbreviations**
B7‐H3: B7 homolog 3
CAFs: cancer‐associated fibroblasts
COAD: colon adenocarcinoma
CTLA‐4: cytotoxic T‐lymphocyte‐associated protein 4
DEGs: differentially expressed genes
DSS: disease‐specific survival
EMT: epithelial–mesenchymal transition
FDR: false discovery rate
FPKM: fragments per kilobase per million
GACs: gastric adenocarcinomas
GSEA: Gene Set Enrichment Analysis
HGF: hepatocyte growth factor
HRs: hazard ratios
ICI: immune checkpoint inhibitors
IHC: immunohistochemistry
K‐M: Kaplan–Meier
MSI: microsatellite instability
NES: normalized enrichment score
OS: overall survival
PD‐1: programmed cell death protein 1
PD‐L1: programmed death ligand 1
PFI: progress‐free interval
ROC: receiver operating characteristic
TCGA: The Cancer Genome Atlas
TTs: tumor tissues

## 1 Introduction

Recent advances in cancer immunotherapy have revolutionized the treatment of advanced cancers, in particular, antibodies targeting programmed cell death 1 (PD‐1), programmed cell death ligand 1 (PD‐L1), and cytotoxic T‐lymphocyte‐associated protein 4 (CTLA‐4) have produced durable remissions in various human solid tumors [1, 2]. Unfortunately, the remarkable therapeutic benefit of anti‐PD‐1/PD‐L1 treatment are only achieved in a minor of patients including high PD‐L1 expression in non‐small cell lung cancer (NSCLC) and a moderate–high tumor mutational burden (TMB). A substantial number of patients do not response to immune checkpoint inhibitor (ICI) [3], which underscore the critical need for biomarkers to predict response to ICI and identify new ICI agents. These findings have garnered our strong interest in immune checkpoints as potential novel therapeutic targets in colon adenocarcinoma. Herein, we performed an explorative analysis of public databases containing data regarding B7‐CD28 family members in colon adenocarcinoma, which is a malignant tumor of the digestive system, ranking third in incidence and second in mortality globally [4] Immune checkpoint molecule B7 homolog 3 (B7‐H3, CD276, or B7RP‐2), a type I transmembrane protein belonging to the Ig superfamily and a member of B7/CD28 family, has immune modulatory functions [5]. The molecular characteristics of its receptor remain still unclear, but overexpression of CD276 is widely observed in numerous human malignancies and seems to be associated with a poorer prognosis [6, 7, 8]. CD276 was initially considered as a co‐stimulatory molecule for T cell activation, nevertheless, but subsequent research revealed that CD276 likely has a predominantly inhibitory role in adaptive immunity, suppressing T‐cell activation and proliferation. Some relevant studies present important evidence for the CD276 are involved in tumor initiation and progression via immunological or non‐immunological mechanisms [9, 10]. Of note, CD276 highly expression might promoted tumor cell growth, invasion, and eventually metastasis, which is proved to be a new independent prognostic indicator for many solid tumors [11]. However, on more in‐depth analyses regarding CD276 expression and its function in colon adenocarcinoma are still lacking; therefore, in the present study, we conducted a comprehensive analysis of CD276 in colon adenocarcinoma.

## 2 Materials and Methods

### 2.1 Data Processing and Ethics Statement

We downloaded high‐throughput sequencing RNA data [fragments per kilobase per million (FPKM) format] and corresponding clinicopathological information from the colon adenocarcinomas project on the TCGA database, which included 481 cases in the cancer group and 41 cases in the normal control group (11:1). Excluding these patients with incomplete clinicopathological information and intact survival data, a total of 453 colon adenocarcinoma patients (COAD = 453) were enrolled. RNA sequencing data were transformed from FPKM format to transcripts per million reads for this study (the conversion method is: converting FPKM to TPM (using the fpkm to Tpm function), then applying log2(TPM + 1) to the TPM values, and using limma for differential analysis). As the TCGA database is open to the public under specific guidelines, it confirms that all written informed consents were obtained before data collection.

### 2.2 Differentially Expressed Genes in Colon Adenocarcinoma

In total, 453 colon adenocarcinoma patients were separated into high‐ and low‐CD276 expression groups according to CD276 median value. The “DESeq2” [12] was used to screen for differentially expressed genes (DEGs) by two‐tailed hypothesis test based on negative binomial generalized linear models, where an adjusted *p* < 0.05 and |log2 fold change (FC)| ≥ 1 were used as a threshold for significance. The results were illustrated in the form of heatmaps and volcano plots by use of the R packages “pheatmap” [13] and “EnhancedVolcano” [14].

### 2.3 Functional Annotation of CD276‐Associated Differentially Expressed Genes in Colon Adenocarcinoma

Gene set enrichment analysis (GSEA) was applied to analyze cancer‐related signaling pathways regulated by CD276 in the TCGA colon adenocarcinoma dataset. Besides, the reference gene sets in this study were the c2.cp.kegg.v7.5.1.symbols.gmt, and the NES (normalized enrichment score) was calculated. If the normal *p* value and FDR (false discovery rate) *q* value were both less than 0.05, the gene set was significantly enriched.

### 2.4 Correlation Analyses for CD276 Expression and Clinicopathological Characteristics of Colon Adenocarcinoma

Differences in the clinicopathological characteristics between high‐ and low‐CD276 expression groups were undertaken using Wilcoxon rank sum test, Pearson's chi‐square test, and Cox regression.

### 2.5 Clinical Significance of CD276 Expression in Colon Adenocarcinoma

CD276 expression was compared between colon adenocarcinoma tumors and pericarcinous tissues by receiver operating characteristic (ROC) analysis to test the predictive value of CD276 for colon adenocarcinoma diagnosis information on colon adenocarcinoma patients' clinical outcome was obtained from TCGA dataset, including overall survival, disease‐specific survival, disease‐free interval, and progression‐free interval. Kaplan–Meier (K‐M) analysis, univariate, and multivariate Cox regression analysis were employed for prognosis analysis. The R package “rms” was used to construct nomograms and calibration plots. The above statistical analyses were all carried out by R (v4.0.5), with *p* values less than 0.05 considered significant.

### 2.6 Investigation of CD276 Biological Function In Vitro

Human normal colonic mucosal epithelial cell line NCM‐460 and cancer cell lines (including SW480, SW620, HCT116, and HT‐29) were obtained from the American Type Culture Collection (ATCC). Cell lines were cultured according to the instructions. The shRNA of CD276 was synthesized by Shanghai GeneChem Co. Ltd. Colon cell lines (including SW480 and HCT116) were transduced with sh‐CD276 lentiviral vectors and selected with puromycin for 10 days. The detailed procedures were referred to the operation manual provided by Shanghai GeneChem Co. Ltd. QT‐PCR and western blotting were applied to verify the knockout efficiency of the lentivirus. The cell viability was detected by using CCK‐8 (Cell Counting Kit‐8). The cell migration and invasion ability were evaluated by scratch assay experiment and transwell migration assay. All experiments above were carried out in three replications and repeated three times.

### 2.7 GEPIA Database Analysis

GEPIA (http://gepia.cancer‐pku.cn/) is a web‐based tool that provides gene expression profiling and interactive analyses based on TCGA data [15]. GEPIA was employed to conduct expression analysis for CD276 in 11 various cancer types. *p* < 0.05 was considered as statistically significant. In addition, expression correlation of CD276 with immune checkpoints in colon adenocarcinoma was also evaluated using the GEPIA database. |*R*| > 0.1 and *p* < 0.05 were set as selection criteria for identifying as statistically significant.

### 2.8 TIMER Database Analysis

Estimates of immune cell infiltration were performed using the TIMER (https://cistrome.shinyapps.io/timer/) web server [16]. We estimated the correlations between CD276 expression and tumor immune‐infiltrating cells in colon adenocarcinoma from TIMER. Statistic *p* < 0.05 was considered as statistically significant.

### 2.9 Statistical Analysis

Differences in the expression of the CD276 in the public data sets were compared by one‐way ANOVA, and differences in clinical information and immune checkpoint inhibitor response between the two different subgroups were compared by the chi‐squared test. Differences in overall survival (OS) and progression‐free interval (PFI) between the two subgroups were compared by the Kaplan–Meier method and log‐rank test. The hazard ratios (HRs) were calculated by univariate Cox regression and multiple Cox regression analysis. GraphPad Prism 8 (GraphPad Software) was used for all the image analysis of this study. All *p* values were two‐sided, with a *p* value of less than 0.05 considered to be statistically significant. R 4.0.4 software was used for some data processing, statistical analysis, and mapping.

### 2.10 Experimental Model and Study Participant Details

Primary tumor and para cancerous tissues of patients with CRC were collected from the Department of Colorectal Surgery, First Hospital of Lanzhou University. Patients (aged 18–75 years) with CRC have no restriction on gender, ancestry, race or ethnicity, or socioeconomic information. None of the enrolled subjects had received radiotherapy or chemotherapy, and had no other cancer co‐morbidities. The study was performed in strict compliance with the Declaration of Helsinki and approved by the Ethics Committee of the First Hospital of Lanzhou University (No. LDYYLL2020‐113).

## 3 Results

### 3.1 CD276 Was Upregulated in Colon Adenocarcinoma Tissues Compared With Normal Colon Tissues

**FIGURE 1:**
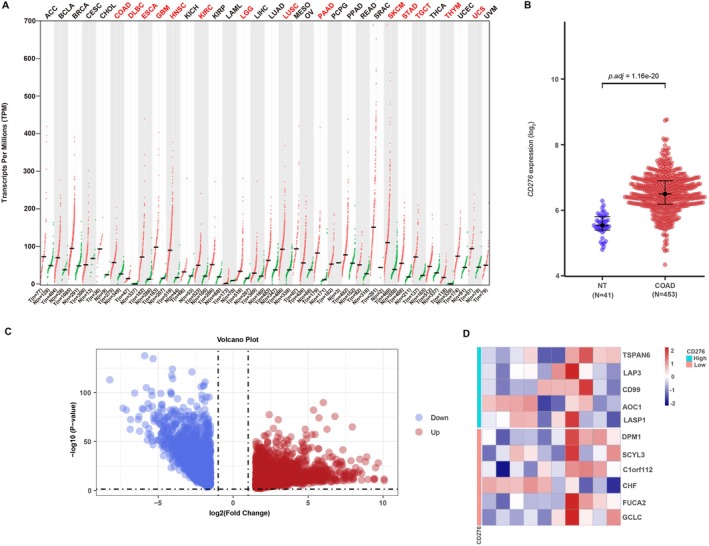
Differential mRNA expression profiles in colon adenocarcinomas (COAD) patients stratified by CD276 levels. (A) The comparison of CD276 expression between tumor and pericarcinous tissue in different types of cancers based on TCGA database. ^ns^ *p* > 0.05; *p < 0.05; **p < 0.01; ***p < 0.001. (B) CD276 expression is higher in COAD tumors than pericarcinous tissue. Based on the median CD276 level, 494 COAD patients from The Cancer Genome Atlas–colon adenocarcinomas project (COAD) Project (TCGA‐COAD) were stratified into high‐ and low‐CD276 expression groups. Shown are expression profiles of mRNA in two groups; and data are presented by volcano plots (C) and heatmaps (D).

Based on TCGA database, we determined the expression of CD276 mRNA in different cancers. As shown in Figure 1A, among 33 cancer types, CD276 was significantly highly expressed in 15 cancers, especially in tumors located in gastrointestinal, urogenital tracts and system of department of gynecology. More specifically, CD276 expression was much higher in colon adenocarcinoma tumors than in pericarcinous tissues (*p* < 0.001, Figure 1B). Interestingly, in none of the investigated cancer profiles was CD276 expression significantly decreased. According to the median expression of CD276 in colon adenocarcinoma, 494 patients were stratified into the high and low‐CD276 expression groups. We next compared mRNA expression between the two groups. Finally, 3833 mRNAs (2093 upregulated and 1740 downregulated, Figure 1C) were recognized as DEGs (absolute value of fold change > 1.5, *p* < 0.05) in the high‐CD276 group. Representative DEGs were also illustrated by heatmaps (Figure 1D).

### 3.2 Functional Annotation of CD276‐Associated Differentially Expressed Genes in Colon Adenocarcinoma

**FIGURE 2:**
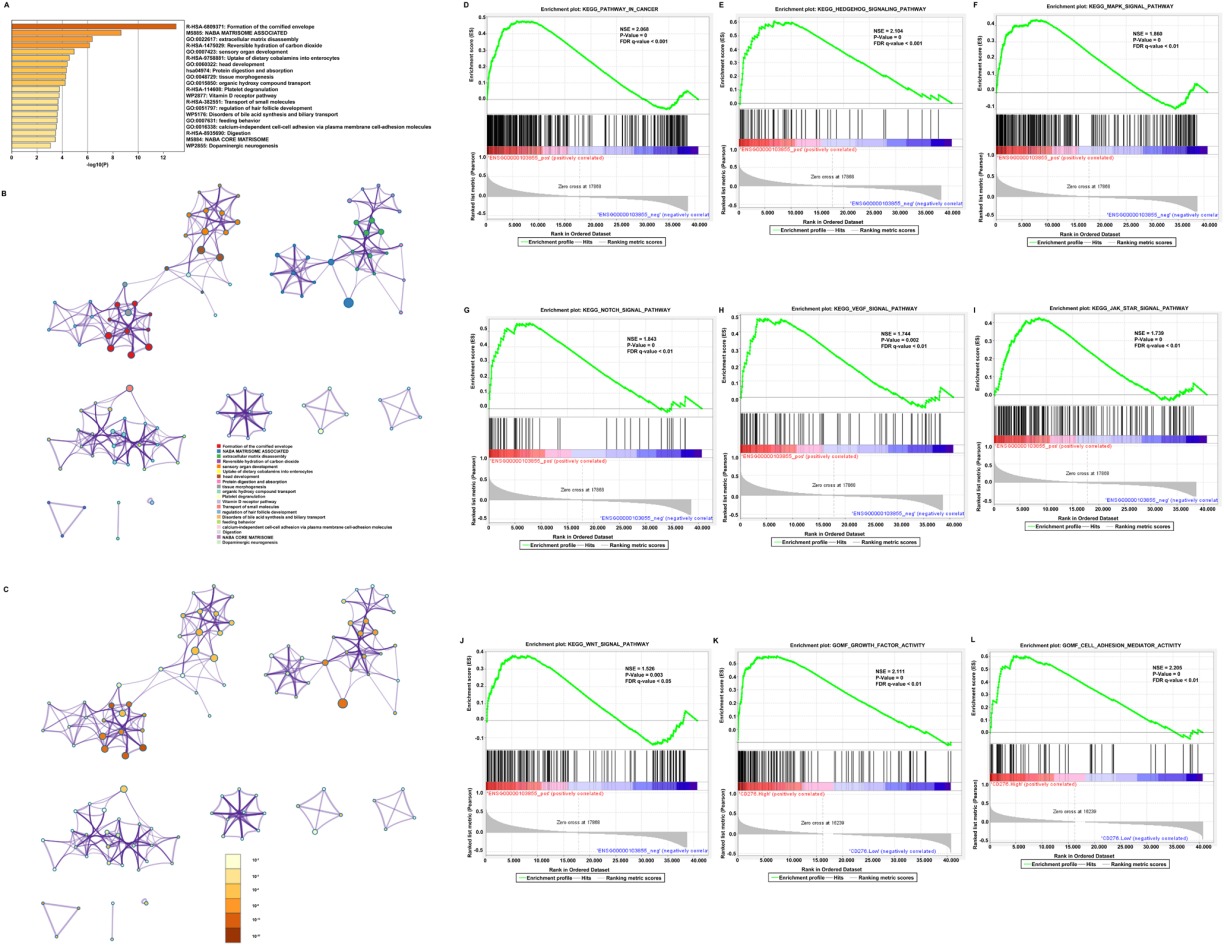
Functional annotation of differentially expressed genes (DEGs) in colon adenocarcinomas (COAD) patients with distinct CD276 levels. According to the Metascape database, 9733 differentially expressed mRNAs between high‐ and low‐CD276 expression groups were used for functional annotation. All statistically enriched terms were identified and then hierarchically clustered into a tree (A) based on the threshold of kappa score as 0.3. Representative terms from the cluster were converted into a network layout (B). The size of a node is proportional to the number of input genes that fall into that term, and the respective color represents its cluster identity. Terms with a similarity score > 0.3 are linked by an edge (the thickness of the edge represents the similarity score). The same enrichment network presents nodes colored by the *p* value (C). (D–L) Representative Gene Set Enrichment Analysis of differentially expressed mRNAs between high‐ and low‐CD276 expression.

In order to evaluate the function of CD276‐associated DEGs in colon adenocarcinoma patients, the software “Metascape” was applied. As presented in Figure 2A–C, we found that several colon adenocarcinoma related pathways were enriched, including extracellular matrix disassembly (GO: 0022617, *p* < 0.001, enrichment factor = 0.243), epithelial to mesenchymal transition in colorectal cancer (WP4239, *p* < 0.001, enrichment factor = 0.873), and Wnt signaling pathway (KEGG hsa04310, *p* = 0.004, enrichment factor = 0.884). Moreover, the GSEA showed CD276‐associated DEGs significantly enriched in cancer pathways (Figure 2D), especially the cell cycle‐related Hedgehog signaling pathway (NES = 2.104, adjusted *p* = 0.000, FDR = 0.0001), MAPK signaling pathway (NES = 1.860, adjusted *p* = 0.000, FDR = 0.0027), Notch signaling pathway (NES = 1.843, adjusted *p* = 0.000, FDR = 0.0034), VEGF signaling pathway (NES = 1.744, adjusted *p* = 0.002, FDR = 0.0080), JAK_STAT signaling pathway (NES = 1.739, adjusted *p* = 0.000, FDR = 0.0081), and WNT signaling pathway (NES = 1.526, adjusted *p* = 0.003, FDR = 0.0441) (Figure 2E–J). More interestingly, CD276‐associated DEGs were associated with the activity of the growth factor and cell adhesion (Figure 2K,L), which are usually involved in oncogenesis.

### 3.3 Association of CD276 Expression and Clinicopathological Characteristics in Colon Adenocarcinoma Patients

**FIGURE 3:**
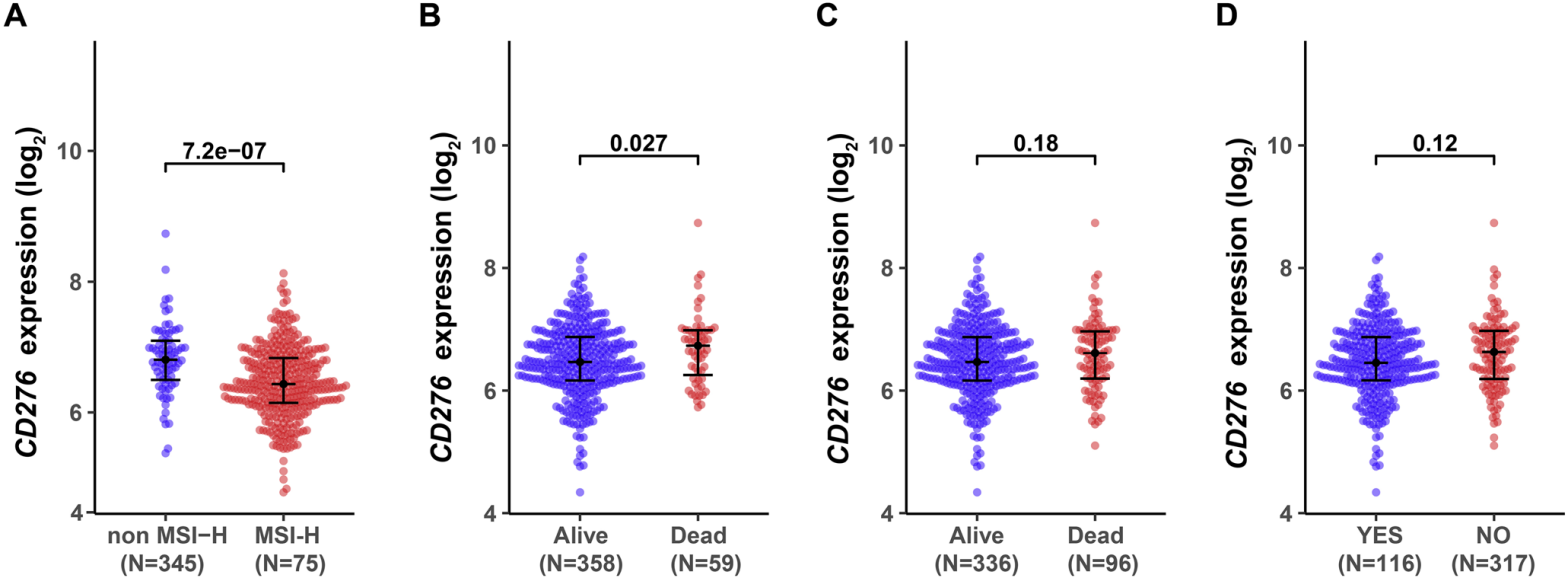
CD276 expression is associated with clinicopathological characteristics in colon adenocarcinomas (COAD) patients. The Wilcoxon rank sum test was applied to analyze the association of CD276 expression with MSI (A), living survival (B), disease‐specific survival (C), and progression‐free interval (D).

We investigated the clinicopathological characteristics of colon adenocarcinoma patients with differential CD276 expression, as shown in Table 1. Compared with the low‐CD276 group, patients in the high‐CD276 group manifested a higher proportion of microsatellite instability high (MSI‐H), death, and progression. However, there was no remarkable distinction with regards to age, gender, TNM stages, CEA level, residual_tumor invasion, lymph node invasion, perineural invasion, radiation therapy, mismatch repair protein (MMR) status, MSI status, KRas, neoadjuvant treatment, or recurrence between high‐CD276 and low‐CD276 groups, which indicated that in colon adenocarcinoma, the expression of CD276 shows no significant correlation with these indicators. Further, we analyzed CD276 expression in patients with different clinicopathological characteristics (Figure 3). CD276 expression was significantly reduced in patients of non‐MSI‐H (Figure 3A) and disease‐specific alive (Figure 3B). As shown in Table 1, patients with progression status (Figure 3C) and alive (Figure 3D) both shared similar CD276 expression levels.

**TABLE 1:** Clinicopathological characteristics of COAD patients with differential CD276 expression.

Characteristics	Total (*N* = 433)	CD276 expression		*p* *
		High (*N* = 130)	Low (*N* = 303)	
Age (year)				
< 65	166 (38.3%)	48 (36.9%)	118 (38.9%)	0.924
≧ 65	267 (61.7%)	82 (63.1%)	185 (61.1%)	
Gender				
Male	232 (53.6%)	66 (50.8%)	166 (54.8%)	0.745
Female	201 (46.4%)	64 (49.2%)	137 (45.2%)	
T stage				
T3–T4	319 (73.7%)	102 (78.5%)	217 (71.6%)	0.622
Tis‐T2	87 (20.1%)	20 (15.4%)	67 (22.1%)	
Unknown	27 (6.2%)	8 (6.2%)	19 (6.3%)	
N stage				
N0	254 (58.7%)	73 (56.2%)	181 (59.7%)	0.82
N1–N2	126 (29.1%)	43 (33.1%)	83 (27.4%)	
Unknown	53 (12.2%)	14 (10.8%)	39 (12.9%)	
M stage				
M0	319 (73.7%)	100 (76.9%)	219 (72.3%)	0.565
M1	48 (11.1%)	16 (12.3%)	32 (10.6%)	
Unknown	66 (15.2%)	14 (10.8%)	52 (17.2%)	
TNM stage				
I	73 (16.9%)	18 (13.8%)	55 (18.2%)	0.983
II	167 (38.6%)	54 (41.5%)	113 (37.3%)	
III	123 (28.4%)	38 (29.2%)	85 (28.1%)	
IV	60 (13.9%)	18 (13.8%)	42 (13.9%)	
Unknown	10 (2.3%)	2 (1.5%)	8 (2.6%)	
CEA level				
Abnormal	98 (22.6%)	27 (20.8%)	71 (23.4%)	0.985
Normal	178 (41.1%)	55 (42.3%)	123 (40.6%)	
Unknown	157 (36.3%)	48 (36.9%)	109 (36.0%)	
Residual_tumor				
R0	313 (72.3%)	100 (76.9%)	213 (70.3%)	0.668
R1–R2	25 (5.8%)	5 (3.8%)	20 (6.6%)	
Unknown	95 (21.9%)	25 (19.2%)	70 (23.1%)	
Vasucular invasion				
Yes	90 (20.8%)	31 (23.8%)	59 (19.5%)	0.901
No	287 (66.3%)	83 (63.8%)	204 (67.3%)	
Unknown	56 (12.9%)	16 (12.3%)	40 (13.2%)	
Lymph_node invasion				
Yes	151 (34.9%)	53 (40.8%)	98 (32.3%)	0.552
No	241 (55.7%)	67 (51.5%)	174 (57.4%)	
Unknown	41 (9.5%)	10 (7.7%)	31 (10.2%)	
Perineural invasion				
Yes	45 (10.4%)	17 (13.1%)	28 (9.2%)	0.831
No	131 (30.3%)	39 (30.0%)	92 (30.4%)	
Unknown	257 (59.4%)	74 (56.9%)	183 (60.4%)	
Radiation therapy				
Yes	9 (2.1%)	1 (0.8%)	8 (2.6%)	0.612
No	354 (81.8%)	104 (80.0%)	250 (82.5%)	
Unknown	70 (16.2%)	25 (19.2%)	45 (14.9%)	
MMR status				
dMMR	55 (12.7%)	20 (15.4%)	35 (11.6%)	0.822
pMMR	267 (61.7%)	80 (61.5%)	187 (61.7%)	
Unknown	111 (25.6%)	30 (23.1%)	81 (26.7%)	
MSI status				
Non MSI‐H	345 (79.7%)	95 (73.1%)	250 (82.5%)	**0.0142** *
MSI‐H	75 (17.3%)	34 (26.2%)	41 (13.5%)	
Unknown	13 (3.0%)	1 (0.8%)	12 (4.0%)	
Kras				
Mut	22 (5.1%)	7 (5.4%)	15 (5.0%)	0.904
Wild	24 (5.5%)	5 (3.8%)	19 (6.3%)	
Unknown	387 (89.4%)	118 (90.8%)	269 (88.8%)	
Neoadjuvant_treatment				
Yes	3 (0.7%)	2 (1.5%)	1 (0.3%)	0.381
No	430 (99.3%)	128 (98.5%)	302 (99.7%)	
Living status				
Alive	337 (77.8%)	90 (69.2%)	247 (81.5%)	**0.0187** *
Dead	96 (22.2%)	40 (30.8%)	56 (18.5%)	
Disease‐specific living status				
Alive	358 (82.7%)	97 (74.6%)	261 (86.1%)	**0.0403** *
Dead	59 (13.6%)	28 (21.5%)	31 (10.2%)	
Unknown	16 (3.7%)	5 (3.8%)	11 (3.6%)	
Recurrence status				
No	163 (37.6%)	40 (30.8%)	123 (40.6%)	0.387
Yes	23 (5.3%)	9 (6.9%)	14 (4.6%)	
Unknown	247 (57.0%)	81 (62.3%)	166 (54.8%)	
Progression status				
No	317 (73.2%)	84 (64.6%)	233 (76.9%)	**0.0302** *
Yes	116 (26.8%)	46 (35.4%)	70 (23.1%)	

Abbreviations: COAD, colon adenocarcinoma; dMMR, MMR absent; MMR, mismatch repair; MSI, microsatellite instability; MSI‐H, MSI‐high; pMMR, MMR proficient.

* Statistically significant *p* values are given in bold, *p* < 0.05.

### 3.4 Predictive Value of CD276 for Colon Adenocarcinoma Patients Diagnosis and Prognosis

**FIGURE 4:**
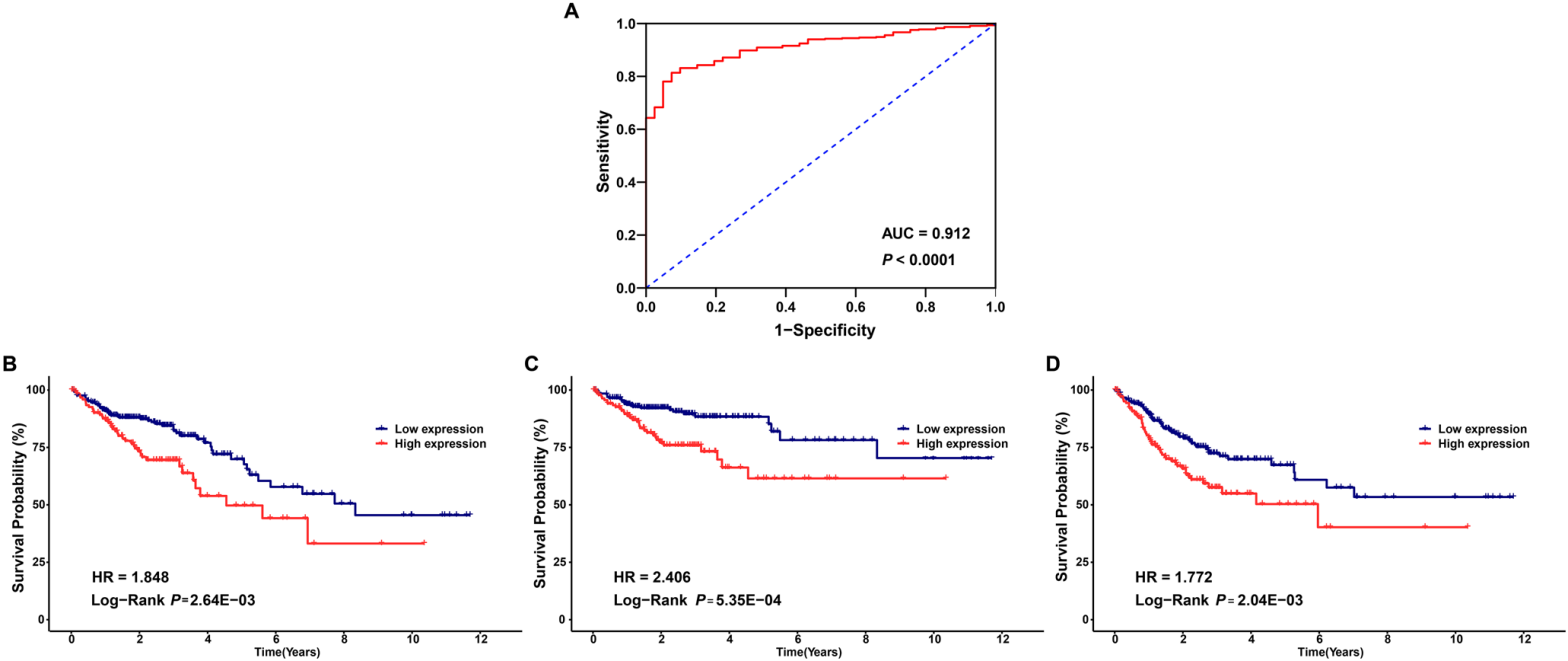
Predictive value of CD276 expression for diagnosis and clinical outcomes in colon adenocarcinomas (COAD) patients. (A) Receiver operating characteristic (ROC) curve analysis evaluating the performance of CD276 for COAD diagnosis. Shown are the Kaplan–Meier analyses comparing overall survival (B), disease‐specific survival (C), and progression‐free interval (D) between high‐ and low‐CD276 expression groups.

To investigate the influence of CD276 on clinical outcomes, a ROC curve was constructed to assess its value on discriminating colon adenocarcinoma diagnosis. As the area under the curve (AUC) was 0.912, CD276 showed significant high sensitivity and specificity for colon adenocarcinoma diagnosis (Figure 4A). Next, K‐M analyses were applied to verify the prediction of CD276 on clinical outcomes. As illustrated in Figure 4B–D, patients in the high CD276 expression group had worse overall survival [hazard ratio (HR): 1.848, *p* = 2.64E−03], disease‐specific survival [hazard ratio (HR): 2.406, *p* = 5.35E−04], and shorter time to progression [hazard ratio (HR): 1.772, *p* = 2.04E−03] than in the low‐CD276 group.

**FIGURE 5:**
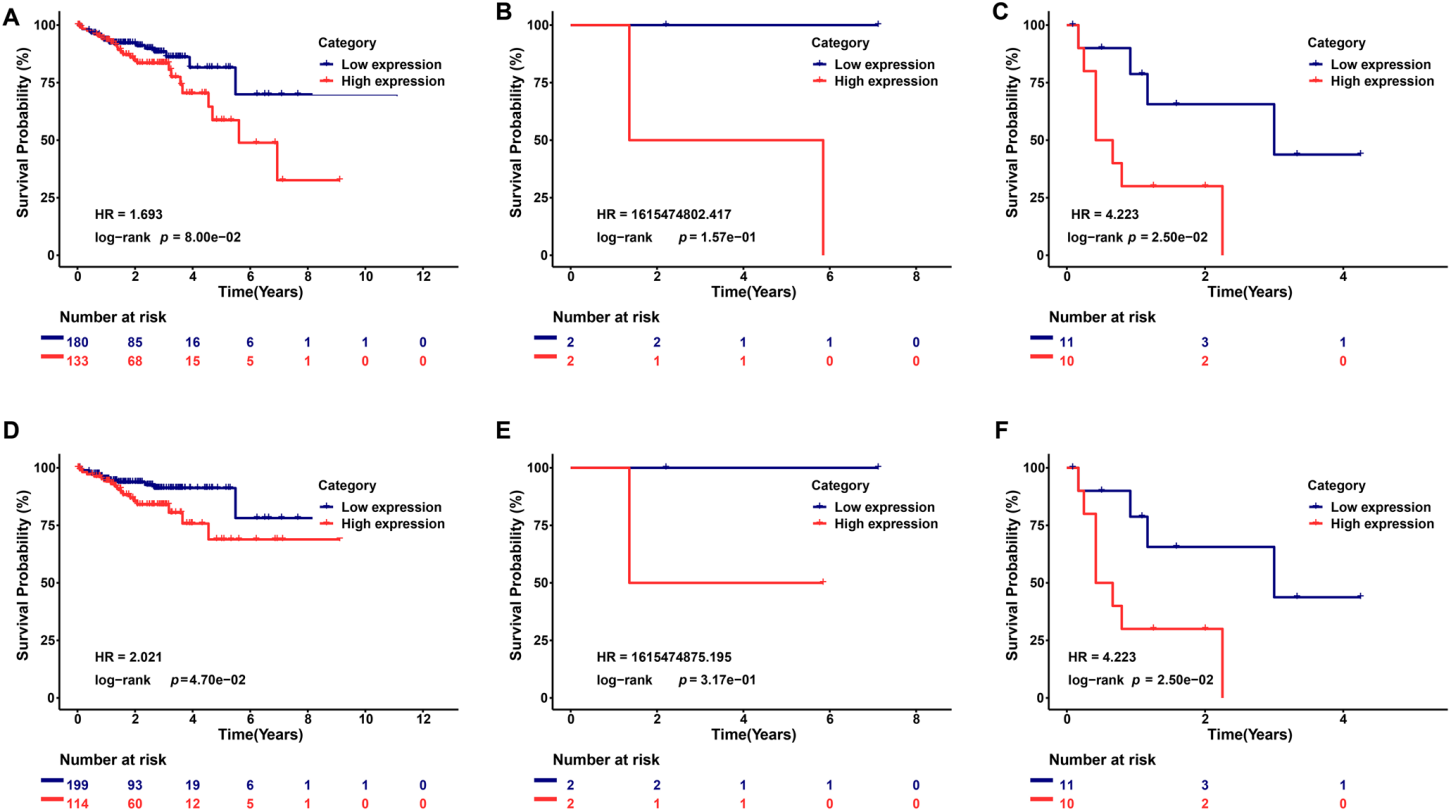
Distinct clinical outcomes based on CD276 expression in colon adenocarcinomas (COAD) patient subgroups. Kaplan–Meier analysis showing the comparison of overall survival (A–C) and disease‐specific survival (D–F) between high‐ and low‐CD276 expression groups in several COAD patient subgroups, including residual_tumor of R0 (A and D), residual_tumor of R1 (B and E), and residual_tumor of R2 (C and F).

Moreover, we performed the Cox regression analysis to further evaluate the predictive value of CD276 on clinical outcomes. As shown in Table 2, in the univariate model, T stage, N stage, M stage, TNM stage, CEA level, Residual_tumor, Vasucular invasion, Lymph_node invasion, MMR status, and CD276 expression were significantly related to overall survival (OS), disease‐specific survival (DSS), and progression‐free interval (PFS) in colon adenocarcinoma (all *p* < 0.05). The following multivariate analyses did not provide any significant predictive ability for OS, DSS, and PFS (all *p* < 0.05) after adjusting other prognostic (Table 2). Conversely, residual_tumor also showed predictive advantages for OS and DSS in multivariate Cox regression analyses. Subsequently, additional K‐M analyses for overall survival and disease‐specific survival in the subgroups based on residual_tumor were also conducted given the heterogeneity between the two groups (Figure 5A–F). For R2 group, all the results demonstrated significantly better clinical outcomes in the low‐CD276 expression groups.

**TABLE 2:** Cox regression analysis for clinical outcomes in COAD patients.

Characteristic	Overall survival, HR (95% CI) *p*				Disease‐specific survival, HR (95% CI) *p*				Progression‐free interval, HR (95% CI) *p*			
	Univariate analysis		Multivariate analysis		Univariate analysis		Multivariate analysis		Univariate analysis		Multivariate analysis	
	HR (95% CI)	*p* *	HR (95% CI)	*p* *	HR (95% CI)	*p* *	HR (95% CI)	*p* *	HR (95% CI)	*p* *	HR (95% CI)	*p* *
Age (≧ 65 vs. < 65)	—	—	—	—	—	—	—	—	—	—	—	—
Gender (female vs. male)	0.89 (0.6, 1.34)	0.584	—	—	0.9 (0.54, 1.51)	0.697	—	—	0.82 (0.56, 1.18)	0.284	—	—
T stage (T3–T4 vs. Tis‐T2)	0.32 (0.14, 0.75)	**0.008**	1.68 (0.15, 19.18)	0.675	0.08 (0.01, 0.56)	**0.011**	0 (0, Inf)	1.000	0.27 (0.12, 0.58)	**0.001**	0.87 (0.1, 7.96)	0.904
N stage (T1–T2 vs. N0)	3.14 (2.04, 4.84)	**< 0.001**	1.26 (0.06, 27.57)	0.884	5.77 (3.12, 10.69)	**< 0.001**	2.21 (0.05, 95.06)	0.679	3.13 (2.12, 4.64)	**< 0.001**	1.8 (0.26, 12.34)	0.548
M stage (M1 vs. M0)	4.79 (2.96, 7.73)	**< 0.001**	5.23 (0.38, 71.81)	0.215	9.6 (5.38, 17.12)	**< 0.001**	18 (0.4, 811.2)	0.137	5.87 (3.8, 9.06)	**< 0.001**	1.29 (0.19,8.84)	0.798
TNM stage (II vs. I)	2.21 (0.77, 6.33)	0.141	0.45 (0.01, 19.87)	0.680	3.46 (0.44, 27.38)	0.239	87 942.45 (0, Inf)	1.000	2.7 (1.06, 6.89)	**0.038**	2.22 (0.09, 53.35)	0.622
TNM stage (III vs. I)	4.28 (1.51, 12.14)	**0.006**	11.12 (0.17, 721.69)	0.258	10.7 (1.43, 80.19)	**0.021**	6 738 319.56 (0, Inf)	1.000	4.33 (1.69, 11.06)	**0.002**	5.52 (0.17, 175.5)	0.333
TNM stage (IV vs. I)	11.28 (3.97, 32.07)	**< 0.001**	5.17 (0.09, 307.23)	0.430	43.94 (5.98, 322.83)	**< 0.001**	1 912 705.7 (0, Inf)	1.000	16.81 (6.61, 42.74)	**< 0.001**	9.71 (0.22, 424.51)	0.238
CEA level (normal vs. abnormal)	0.34 (0.2, 0.6)	**< 0.001**	1.85 (0.45, 7.53)	0.392	0.36 (0.18, 0.69)	**0.002**	5.22 (0.79, 34.51)	0.086	0.36 (0.23, 0.58)	**< 0.001**	0.82 (0.31, 2.2)	0.692
Residual_tumor (R0 vs. R1–R2)	4.94 (2.7, 9.03)	**< 0.001**	5.76 (1.36, 24.3)	**0.017**	6.74 (3.52, 12.92)	**< 0.001**	42.39 (3.4, 528.75)	**0.004**	4.79 (2.81, 8.19)	**< 0.001**	2.64 (0.76, 9.16)	0.126
Vascular invasion (yes vs. no)	0.38 (0.24, 0.59)	**< 0.001**	0.87 (0.24, 3.19)	0.831	0.25 (0.14, 0.43)	**< 0.001**	1.67 (0.26, 10.6)	0.586	0.34 (0.22, 0.5)	**< 0.001**	0.5 (0.19, 1.35)	0.172
Lymph_node invasion (yes vs. no)	2.25 (1.46, 3.47)	**< 0.001**	0.63 (0.17, 2.34)	0.495	3.77 (2.11, 6.74)	**< 0.001**	0.86 (0.12, 6.21)	0.878	2.37 (1.6, 3.49)	**< 0.001**	0.59 (0.18, 1.97)	0.392
Perineural invasion (yes vs. no)	1.89 (0.96, 3.71)	0.066	—	—	2.8 (1.25, 6.26)	**0.012**	0.52 (0.07, 3.72)	0.514	2.28 (1.24, 4.2)	**0.008**	2.25 (0.61, 8.24)	0.222
Radiation therapy (yes vs. no)	0.63 (0.09, 4.54)	0.646	—	—	0.87 (0.12, 6.3)	0.890	—	—	1.52 (0.48, 4.81)	0.477	—	—
MMR status (dMMR vs. pMMR)	5.67 (1.38, 23.36)	**0.016**	4.97 (0.81, 30.63)	0.084	4.5 (1.08, 18.65)	**0.038**	3.01 (0.3, 30.09)	0.348	1.45 (0.77, 2.75)	0.253	—	—
MSI status (MSI‐H vs. non MSI‐H)	0.85 (0.5, 1.46)	0.568	—	—	0.74 (0.37, 1.51)	0.414	—	—	0.75 (0.45, 1.25)	0.268	—	—
Kras (Mut vs. Wild)	0.8 (0.34, 1.85)	0.599	—	—	0.67 (0.18, 2.5)	0.550	—	—	1.31 (0.56, 3.05)	0.528	—	—
Neoadjuvant_treatment (yes vs. no)	3.67 (0.51, 26.53)	0.198	—	—	4.99 (0.69, 36.38)	0.112	—	—	2.4 (0.33, 17.25)	0.385	—	—
CD276 (high vs. low)	1.02 (1, 1.04)	**0.031**	1.04 (0.98, 1.11)	0.192	1.04 (1.01, 1.06)	**0.002**	1.06 (0.97, 1.15)	0.221	1.03 (1.01, 1.05)	**0.004**	1.01 (0.96, 1.06)	0.721

Abbreviations: CI, confidence interval; COAD, colon adenocarcinoma; dMMR, MMR absent; HR, hazard ratio; MMR, mismatch repair; MSI, microsatellite instability; MSI‐H, MSI‐high; pMMR, MMR proficient.

* Statistically significant *p* values are given in bold, *p* < 0.05.

### 3.5 Validation of the Prognostic Value of CD276 in Colon Adenocarcinoma Based on Nomograms

**FIGURE 6:**
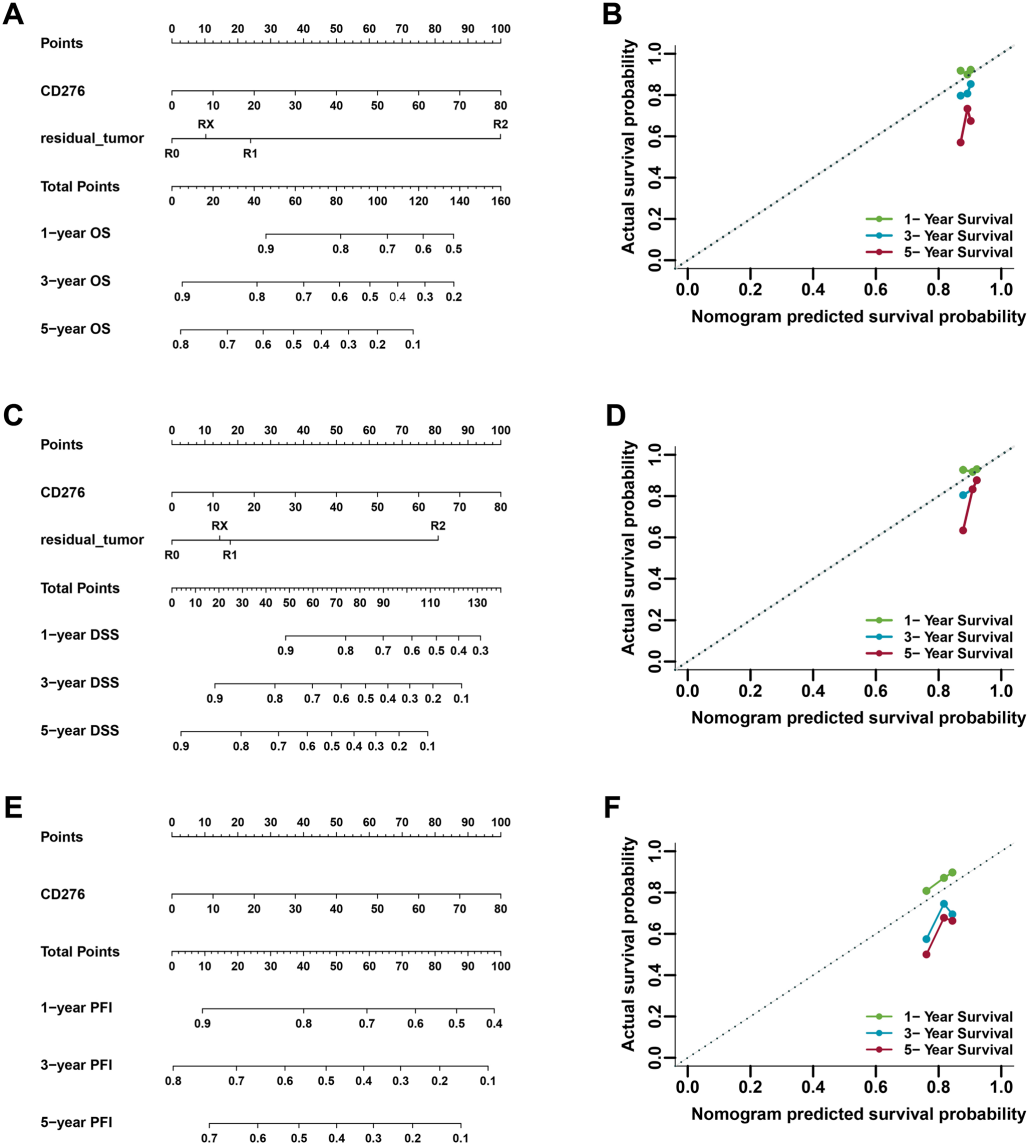
Construction and validation of nomograms based on CD276 expression. Shown are the nomograms constructed to establish CD276 expression‐based risk scoring models for 1‐, 3‐, and 5‐year overall survival (A), disease‐specific survival (C), and progression‐free interval (E). Calibration plots validating the efficiency of nomograms for overall survival (B), disease‐specific survival (D), and progression‐free interval (F). DSS, disease‐specific survival; OS, overall survival; PFI, progression‐free interval.

To further validate the prognostic value of CD276, nomograms were constructed based on CD276 mRNA expression and residual_tumor, which were identified as independent indicators in terms of OS and DSS via multivariate analyses. A calibration curve was drawn to test the efficiency of the nomogram. Residual_tumor, as well as CD276, were included in the nomogram to predict overall survival, which had a C‐index of 0.543 (Figure 6A). Residual_tumor and CD276 were also evaluated in a nomogram as predictors of disease‐specific survival, with a C‐index of 0.556 (Figure 6C). A predictive nomogram for progression‐free interval was constructed using only CD276 with a C‐index of 0.570 (Figure 6E). The calibration curves all presented desirable prediction of the three nomograms for the 1‐, 3‐, and 5‐year clinical outcomes, with the exception of the 1‐year prediction for clinical outcomes, which was accurately estimated (Figure 6B,D,F).

### 3.6 CD276 Positively Correlates With Immune Cell Infiltration in Colon Adenocarcinoma

**FIGURE 7:**
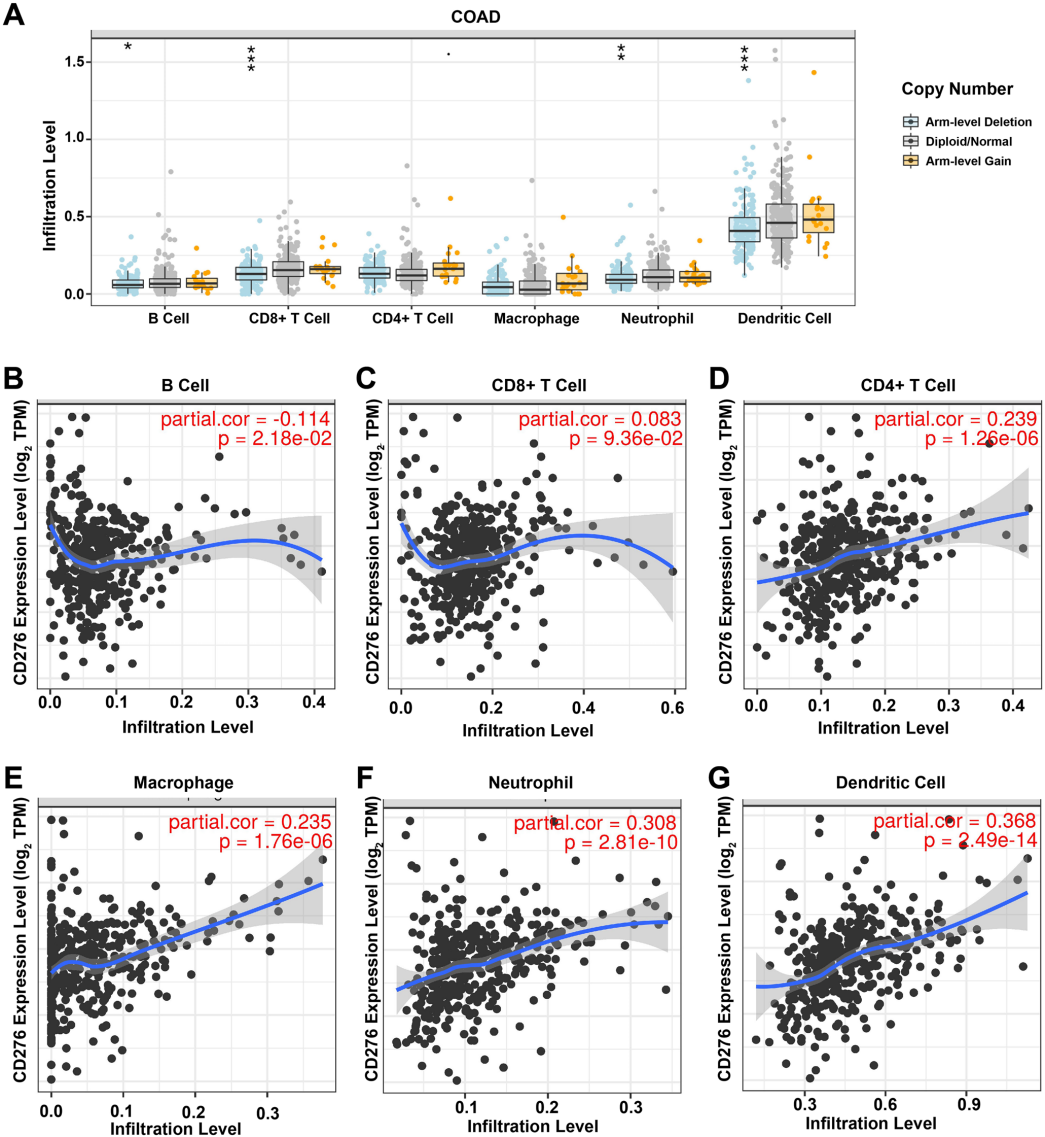
The relationship of immune cell infiltration with CD276 level in COAD (A) The infiltration level of various immune cells under different copy numbers of CD276 in COAD. (B–G) The correlation of CD276 expression level with B‐cell (B), CD8+ T‐cell (C), CD4+ T cell (D), macrophage (E), neutrophil (F), or dendritic cell (G) infiltration level in COAD.

CD276 plays a critical role in the regulation of immune responses from CD28:B7 immunoglobulin family. As shown in Figure 7A, significant changes of B cells, CD8+ T cells, neutrophils, and dendritic cells were detected under various copy numbers of CD276 in colon adenocarcinoma. Correlation analysis could provide key clues for the studies of CD276 function and mechanism. Whereas, our analysis of human colon adenocarcinoma has suggested a correlation between CD276 and immune cell infiltrates. As presented in Figure 7B–G, TIMER revealed a significant correlation between CD276 and immune cell infiltrations, including B cells, CD8+ T cells, CD4+ T cells, macrophages, neutrophils, and dendritic cells in colon adenocarcinoma.

### 3.7 Expression Correlation of CD276 and Biomarkers of Immune Cells in Colon Adenocarcinoma

To determine if CD276 in plays an important role in tumor immune, we detected the correlation between CD276 and immune cell biomarkers in colon adenocarcinoma by GEPIA database. As listed in Table 3, CD276 correlated significantly with the measured biomarkers of immune cells, including B cell's biomarkers (CD79A), CD8+ T cell's biomarkers (CD8A and CD8B), CD4+ T cell's biomarker (CD4), M1 macrophage's biomarkers (IRF5 and PTGS2), M2 macrophage's biomarkers (CD163, VSIG4, and MS4A4A), neutrophil's biomarkers (ITGAM and CCR7), and dendritic cell's biomarkers (HLA‐DPB1, HLA‐DQB1, HLA‐DRA, HLA‐DPA1, CD1C, NRP1, and ITGAX) in colon adenocarcinoma. These findings partially support that CD276 is positively linked to immune cell infiltration.

**TABLE 3:** Correlation analysis between CD276 and biomarkers of immune cells in COAD determined by GEPIA database.

Immune cell	Biomarker	*R* value	*p* value
B cell	CD19	0.079	0.11
	CD79A	0.13^a^	0.032* ^a^
CD8+ T cell	CD8A	0.13^a^	0.027* ^a^
	CD8B	0.13^a^	0.038* ^a^
CD4+ T cell	CD4	0.51^a^	0*** ^a^
M1 macrophage	NOS2	−0.015	0.8
	IRF5	0.18^a^	0.0035** ^a^
	PTGS2	0.29^a^	6.8e−07*** ^a^
M2 macrophage	CD163	0.56^a^	0*** ^a^
	VSIG4	0.53^a^	0*** ^a^
	MS4A4A	0.55^a^	0*** ^a^
Neutrophil	CEACAM8	0.029	0.63
	ITGAM	0.61^a^	0*** ^a^
	CCR7	0.2^a^	0.00063*** ^a^
Dendritic cell	HLA‐DPB1	0.33^a^	1.4e−08*** ^a^
	HLA‐DQB1	0.22^a^	0.00025*** ^a^
	HLA‐DRA	0.34^a^	1e−08*** ^a^
	HLA‐DPA1	0.27^a^	6.6e−06*** ^a^
	CD1C	0.25^a^	2.2e−05*** ^a^
	NRP1	0.7^a^	0*** ^a^
	ITGAX	0.56^a^	0*** ^a^

*Note:* a = these results are statistically significant. *p < 0.05; **p < 0.01; ***p < 0.001.

### 3.8 Relationship Between CD276 and Immune Checkpoints in Colon Adenocarcinoma

**FIGURE 8:**
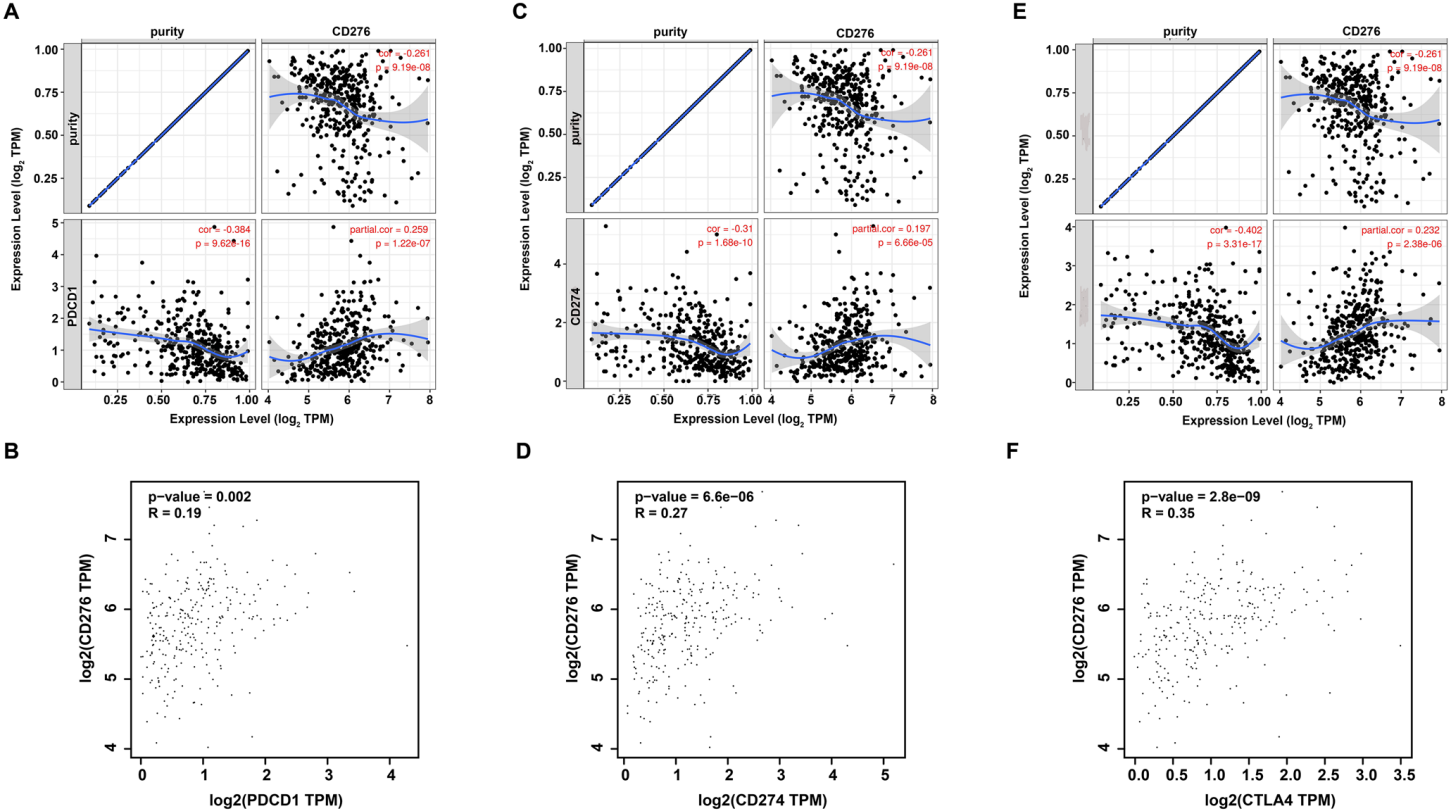
Correlation of CD276 expression with PD‐1, PD‐L1, and CTLA‐4 expression in COAD. (A) Spearman correlation of CD276 with expression of PD‐1 in COAD adjusted by purity using TIMER. (B) Spearman correlation of CD276 with expression of PD‐L1 in COAD adjusted by purity using TIMER. (C) Spearman correlation of CD276 with expression of CTLA‐4 in COAD adjusted by purity using TIMER. (D) The expression correlation of CD276 with PD‐1 in COAD determined by GEPIA database. (E) The expression correlation of CD276 with PD‐L1 in COAD determined by GEPIA database. (F) The expression correlation of CD276 with CTLA‐4 in COAD determined by GEPIA database.

PD1/PD‐L1 and CTLA‐4 are important immune checkpoints that are responsible for tumor immune escape. Considering CD276 has the potential oncogenic role in colon adenocarcinoma, we further explore the relationship of CD276 with PD1, PD‐L1, or CTLA‐4. As suggested in Figure 8A–C, CD276 expression was significantly positively correlated with PD1, PD‐L1, and CTLA‐4 in colon adenocarcinoma, which was adjusted by purity. Similar to TIMER data analysis, we also found that there was a significant positive correlation of CD276 with PD1, PD‐L1, or CTLA‐4 in colon adenocarcinoma (Figure 8D–F). These results demonstrate that tumor immune escape might be involved in CD276‐mediated carcinogenesis of colon adenocarcinoma.

### 3.9 Knockdown of CD276 Expression Reduced Cell Proliferation, Migration, and Invasion of Colon Adenocarcinoma Cells

**FIGURE 9:**
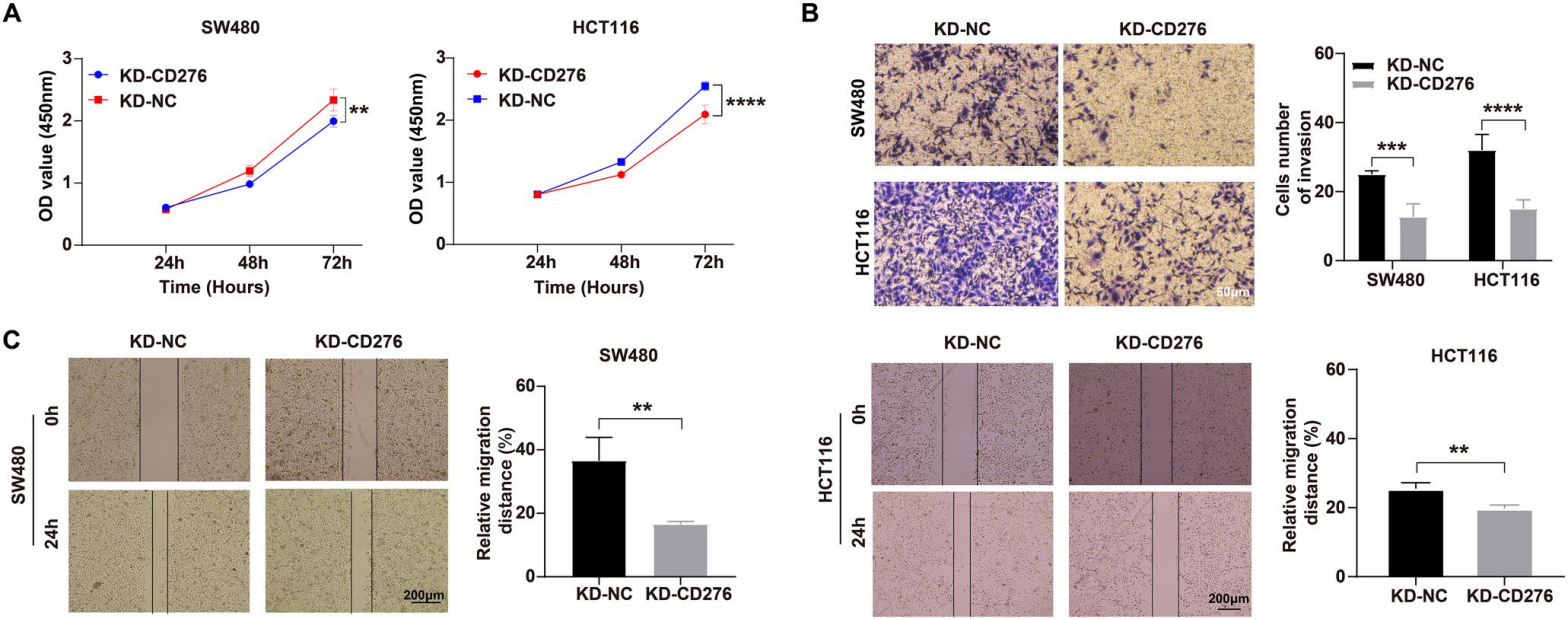
Validation the function of CD276 with in vitro experiments by SW480 and HCT116 cell lines. (A) Cell proliferation of SW480 and HCT116 after being transfected with NC and CD276 lentiviral‐shRNA vector, *p < 0.05, **p < 0.01. (B) Wound healing of SW480 and HCT116 after being transfected with NC and CD276 lentiviral‐shRNA vector, *p < 0.05, **p < 0.01. (C) Cell invasion and invasion of SW480 and HCT116 after being transfected with NC and CD276 lentiviral‐shRNA vector, ***p < 0.001, ****p < 0.0001.

Firstly, we knocked down the CD276 protein level by shCD276 in both SW480 and HCT116 cell lines, which obtained the higher level of CD276 as compared to control NCM‐460 and confirmed the knockdown by western blot (WB). Compared with SW480/sh‐NC cells, the cell viability of SW480/sh‐CD276 cells at 48 and 72 h was significantly decreased. The same trend was observed in HCT116 cells (Figure 9A). Cell migration and cell invasion assays were also performed to compare the cell adhesion and migration; wound healing assay showed that the knockdown of CD276 suppressed significantly the migration of SW480 and HCT116 cells (Figure 9B). Furthermore, we also evaluated the impact of cell invasion in sh‐NC and sh‐CD276 subgroups. The results showed that knockdown of CD276 can decrease about 10%–20% cell invasion (Figure 9C); therefore, the target therapy to CD276 might be a potential new strategy for colon adenocarcinoma patients.

### 3.10 CD276 Expression Elevated in Tumor Tissues as Compared With Normal Tissues

**FIGURE 10:**
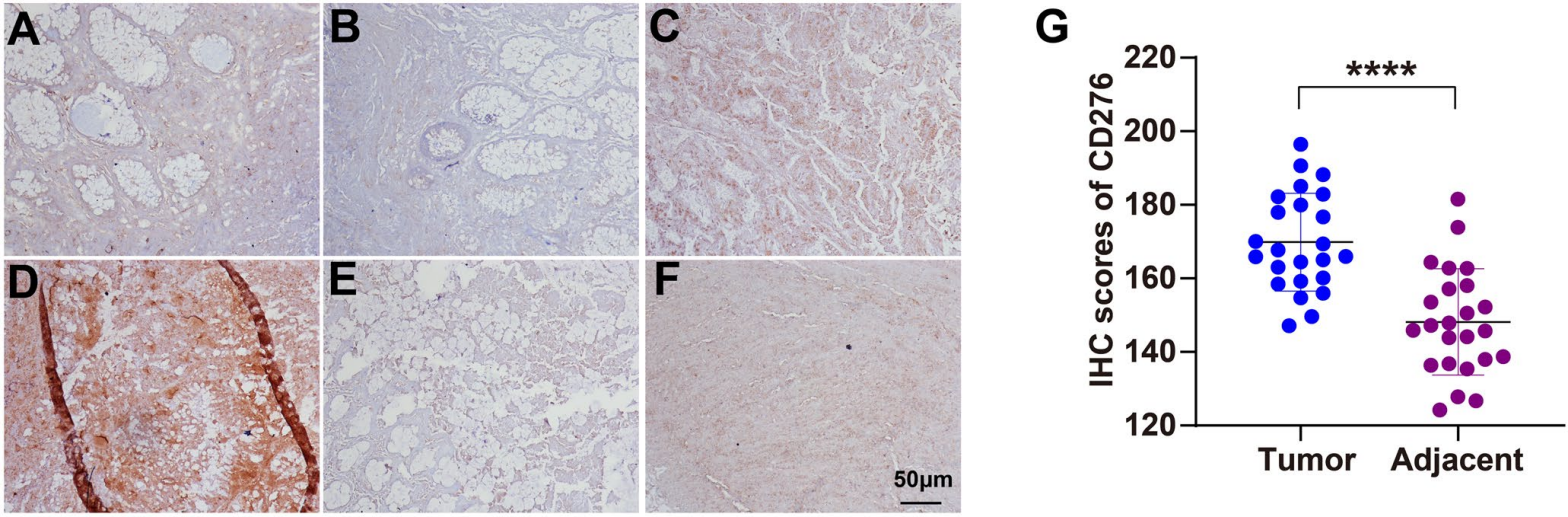
Protein expression profile of CD276 in COAD (200×) (A–D) and normal colon tissue (100×) (E and F). Statistical result of the total score of CD276 immunohistochemical (IHC) staining analyzed by two senior pathologists in tumor and adjacent tissues (G). ****p < 0.0001.

In order to further explore our findings from TCGA, we examined the expression of CD276 by immunohistochemistry (IHC) in 26 paired colon adenocarcinoma tumors and para‐tumor tissue samples. The results showed that CD276 staining in colon adenocarcinoma tumor tissues (TTs) was mainly located in the cell membrane, and the corresponding para‐tumor tissues showed extremely weak staining. In contrast, CD276 expression was relatively high in colon adenocarcinoma TTs; among the colon adenocarcinoma TT samples, three had negative staining (15.1%, Figure 10A), six had weak positive staining (23.9%, Figure 10B), 10 had moderate positive staining (42.8%, Figure 10C), and seven had strong positive staining (18.2%, Figure 10D). Overall, the B7‐H3 expression levels of nontumor tissue samples (Figure 10E,F) were significantly lower than those of their paired colon tumor tissue samples (*p* < 0.0001, Figure 10G).

## 4 Discussion

In the present study, we investigated expression profiles, the association of clinicopathological parameters, and the clinical significance of an important immune checkpoint factor, CD276, by analyzing the colon adenocarcinoma dataset from TCGA. In colon cancer, we first observed prominent increased CD276 expression compared with that in normal tissue. In our study, we further found that CD276 expression was upregulated in the colon adenocarcinoma tissues at the protein levels from patients' samples, and IHC staining revealed CD276 protein expression in most of the colon adenocarcinoma samples. Differentially expressed genes (DEGs) regulated by higher CD276 levels were specifically enriched in cell adhesion‐ and growth factor‐associated pathways, as well as signal pathways in cancer. We also revealed a novel association of CD276 expression with MSI status. Our reports showed the prognostic value of CD276 for overall survival, disease‐specific survival, and progression‐free interval in colon adenocarcinoma patients. Finally, we also conducted the knockdown studies of CD276 in two colon adenocarcinoma cell lines, SW480 and HCT116. Results showed in colon tumor, CD276 knockdown not only inhibit tumor cell migration/invasion, but also cell proliferation. Steven S et al. [17] found that tumor cells and tumor vessels frequently expressed high levels of CD276. Nevertheless, our results are in line with the findings of Steven S et al. who suggested that high CD276 levels correlate with poor prognosis, increased pathological grade and metastasis, drug resistance, and short survival. Zhan et al. [18] indicated that CD276 was aberrantly expressed not only on tumor cells but also on adjacent stromal cells of gastric adenocarcinomas (GACs); inhibition of CD276 via small interfering RNA leads to reducing the potency of invasion and migration in cancer‐associated fibroblasts (CAFs) and suppression of cytokine secretion.

Further, we attempted to delineate the biological significance and mechanism through which CD276 is involved in colon cancer. Studies have found that the downregulation of CD276 lead to a reduced cell adhesion to fibronectin and the capacity of cells to migrate and invade the matrigel in vitro. However, since the matrigel invasion capacity of these cells was significantly decreased by CD276 silencing, the symptom‐free survival time of mice and rats was prolonged after injection with CD276‐knockout cells [19]. Study reported that CD276 stimulated CAFs secrete hepatocyte growth factor (HGF), which mediate tumor cell proliferation, invasion, and migration [20]. In our study, based on functional annotation of CD276‐associated DEGs, the activity of the growth factor and cell adhesion were closely associated with CD276 expression. Moreover, CD276 was associated with epithelial–mesenchymal transition (EMT) in colon adenocarcinoma. Based on additional GSEA, several cancer‐related pathways were enriched in the high‐CD276 group. The above data all provided evidence that CD276 functions as a critical cancerogenic factor in colon adenocarcinoma. CD276 activates ROS/Src/c‐Cbl signal pathway in multiple myeloma by integrating redox regulation and persistently activates JAK2/STAT3 at the level of STAT3 suppressor degradation [21]. CD276 promotes metastasis of breast cancer to lung via activation of Raf/MEK/ERK/MAPK cascade [22]. CD276 induces VEGFA expression and promotes angiogenesis in colorectal cancer through activation of the NF‐kappaB pathway [23]. Therefore, CD276 expression might be of great importance in colon adenocarcinoma tumorigenesis by affecting function of cancer‐associated signal pathways.

However, the clinical significance of CD276 in colon adenocarcinoma is another issue of great interest. The high diagnostic value of CD276 was demonstrated by a ROC curve with an AUC of 0.912 for colon adenocarcinoma. Because the existing serum biomarkers lacked high sensitivity and specificity for the surveillance of metastatic or recurrent disease in colon adenocarcinoma, it may be significant to determine the cut‐off value of CD276 and use as a biomarker for early alarming tumor recurrence after surgery [24, 25, 26]. In addition, the clinical implication of CD276 upregulation in colon adenocarcinoma was evaluated. Our study demonstrated that high CD276 expression was strongly associated with MSI status, death, disease‐specific survival, and progression‐free interval, highlighting that CD276 might lead to the progression and poor prognosis of colon adenocarcinoma. Meanwhile, KM analyses showed that colon adenocarcinoma patients with high CD276 expression had relatively poor OS, DSS, and PFI (all *p* < 0.05). So far, significant heterogeneity in prognostic factors has been considered among individuals with colon adenocarcinoma and the existing clinical prognostic predictors are not precise enough [27, 28], it is extremely necessary to establish a genomic–clinicopathologic nomogram to predict the long‐term outcomes of patients with advanced colorectal cancer. We created nomograms models with the independent prognostic parameters (including CD276 expression and residual_tumor) for OS and DSS of patients. The results showed that the genomic–clinicopathologic nomograms represented more precise prognostic models compared with the other clinical predictors alone. Our analysis has important implications that CD276 will be clinically useful for monitoring postoperative recurrence and guiding treatment decision making.

A multitude of studies have confirmed that the combination of immunotherapy with chemotherapy and radiotherapy could recruit tumor infiltrating immune cell into the TME, protect the body from tumor reattack and ultimately prolong the survival of patients [29, 30, 31]. Our work confirmed that CD276 was positively correlated with various immune cells, including B cell, CD8+ T cell, CD4+ T cell, macrophage, neutrophil, and dendritic cell in colon adenocarcinoma. Moreover, CD276 was also markedly positively associated with biomarkers of these infiltrated immune cells. These findings indicated that tumor immune infiltration might partially account for CD276‐mediated oncogenic roles in colon adenocarcinoma. Also, the immune cells infiltration and the expression of immune checkpoints in tumor microenvironment correlate with response to immunotherapy [32, 33]. Thus, we also assessed the relationship between CD276 and immune checkpoints. The results demonstrated that high expression of CD276 was strongly linked to PD1, PD‐L1, or CTLA‐4 in COAD.

It is well‐known that the molecular expression of immune checkpoint such as CTLA‐4, PD‐1, PD‐L1, PD‐L2, LAG‐3, IDO1, and IL‐10 could impact the infiltration of immune cells in the tumor microenvironment, thereby regulating the immune state of the tumor microenvironment [34, 35, 36]. Several promising results have been achieved with checkpoint inhibitors such as nivolumab, pembrolizumab, and ipilimumab, resulting in U.S. FDA's approval for the treatment of patients with MSI‐H advanced colon adenocarcinoma [37, 38, 39]. Our results revealed that abnormal CD276 expression in colon adenocarcinoma patients was closely correlated with distinct patterns of MSI status. These findings provide evidence of a potential mechanistic link between MSI, the immunocyte infiltration, immunotherapy, and increased CD276 expression, and the combination therapy of anti–CD276 monoclonal antibodies with other immune checkpoint inhibitors may yield superior curative effects for colon adenocarcinoma patients in future. Based on the above findings, several preclinical studies revealed that cotargeting CD276 and PD‐L1 molecules may improve the antitumor response by direct killing and acting on both the innate and adaptive immune systems [40, 41]. Targeting CD276 in combination with other immune checkpoints such as PD‐1, PD‐L1 or CTLA‐4, or therapeutic antibody–drug conjugates (ADCs) targeting B7‐H3 or PD‐L1 have demonstrated efficacy against tumors and manageable safety profiles in clinical settings [42, 43]. Combining these findings, immunomodulating bispecific antibody–drug conjugates (BsADC) based on the B7‐H3 × PD‐L1–targeting BsAb skeleton may hold promise. However, we should pay more attention to the toxic side effects and corresponding strategies when applying different immune checkpoint inhibitors for colon adenocarcinoma patients and special high‐risk colorectal cancer patients [44, 45].

Although we uncovered a potential mechanism for CD276 activity in colon adenocarcinoma tumorigenesis and its predictive value in colon adenocarcinoma clinical outcomes, our study still presented several limitations. First, because of the incomplete information about treatments and corresponding responses, we could not evaluate a specific role for CD276 in colon adenocarcinoma treatment. Second, we mainly focused on the RNA sequencing data from the TCGA database and information on the relative protein levels or downstream pathways of CD276 was lacking. Third, the information from TCGA database has limited sample sources and representativeness, and its data updates are slow with incomplete coverage. Thus, further studies will focus on the direct mechanism of CD276 as an oncogenic factor leading to the development and progression of colon adenocarcinoma. Additionally, findings from the above basic research need to be validated in independent clinical cohorts to enhance the reliability of the evidence.

## 5 Conclusion

In this study, we found a certain association between CD276 and colon adenocarcinoma, suggesting that CD276 may play an important role in colon cancer pathogenesis. Moreover, the overexpression of CD276 is also highly correlated with some biomarker genes in immune‐infiltrating cells (such as CD79A, CD8, IRF5, CD163, and HLA), which may also become a new research target in the future. However, our results were obtained through various bioinformatics analyses and simple tests in vitro, which can only provide some preliminary theoretical basis. Future research based on large samples needs to be carried out to clarify the biological functions of CD276 and its impact on immune response.

## Author Contributions

Rui Chen and Fei Su were responsible for the conception and design. Tao Zhang and Fei Su were responsible for the administrative support. Fei Su and Jingru Yang were responsible for the provision of study materials or patients. Longxia Zhang and Tao Zhang were responsible for the collection and assembly of data. Rui Chen and Fei Su were responsible for the data analysis and interpretation.

## Funding

This study was supported by the Natural Science Foundation of Gansu Province (Grant 25JRRA1001) and the Lanzhou University National Innovation Project (Grant 20240060048).

## Ethics Statement

The authors have nothing to report.

## Consent

Samples were collected after obtaining informed consent from all donors.

## Conflicts of Interest

The authors declare no conflicts of interest.

## Data Availability

The data that support the findings of this study are available from the corresponding author upon reasonable request.
